# Risk factors for endophthalmitis requiring evisceration or enucleation

**DOI:** 10.1038/srep28100

**Published:** 2016-06-15

**Authors:** Xuehui Lu, Danny Siu-Chun Ng, Kangkeng Zheng, Kun Peng, Chuang Jin, Honghe Xia, Weiqi Chen, Haoyu Chen

**Affiliations:** 1 Joint Shantou International Eye Center, Shantou University and the Chinese University of Hong Kong, Shantou, China; 2 Department of Ophthalmology & Visual Sciences, the Chinese University of Hong Kong, Hong Kong, China

## Abstract

Endophthalmitis has devastating sequelae resulting in blindness and even loss of eyeball. Although the prognosis of endophthalmitis has much improved with the advances of antibiotics and vitreoretinal surgery, of the number of patients that required evisceration or enucleation is still significant. We retrospectively reviewed the charts of 210 eyes of 210 patients with endophthalmitis andcompared the group that required evisceration or enucleation with those that received salvaging therapies. Regression analysis was used to identify the risk factors for evisceration or enucleation. Thirty eyes (14.3%) underwent enucleation or evisceration. The group of eviscerated or enucleated eyes were older (58.7 vs. 42.2 years, p < 0.001), had more women (56.7% vs. 22.2%, p = 0.003), had poorer initial visual acuity (2.79 vs. 2.10 LogMAR, p < 0.001), and had longer duration before intervention (18.03 vs. 5.74 days, p = 0.031). The most common primary indications for endophthalmitis were infections from corneal ulcer (50.0% vs. 4.4%, p < 0.001) andfrom endogenous source (23.3% vs. 5.6%, p < 0.001). Less common indications were trauma (26.7% vs. 67.8%, p < 0.001) and postoperative (6.7% vs. 22.2%, p = 0.049) endophthalmitis. After adjusting for confounding factors, corneal ulcer-related endophthalmitis, endogenous endophthalmitis and initial visual acuity were the independent risk factors for evisceration or enucleation.

Endophthalmitis is an inflammatory condition of the intraocular structure commonly caused by infection. The source of infection can be exogenous or endogenous. Common causes of exogenous endophthalmitis are trauma, intraocular surgery or infective keratitis. Endophthalmitis has devastating sequelae resulting in blindness and even loss of the eyeball. Even with the advances of antibiotics and vitreoretinal surgery which improved the prognosis of endophthalmitis, there remains a significant number of patients requiring evisceration or enucleation because antibiotics or vitrectomy failed to control infection or because of severe intraocular tissue destruction.

Evisceration or enucleation is often indicated in eyes that failed to recover from various interventional attempts. A report in 2005 showed the common indications of enucleation were trauma (36%), malignant tumor (20.7%), glaucoma (19.6%), phthisis bulbi (9%) and endophthalmitis (8.1%)^1^. Another report in 2008 showed that the most frequent indications of enucleation or evisceration were trauma (32.6%), following by glaucoma (27.6%), endophthalmitis (27.3%) and tumor (12.6%)^2^. These two studies revealed that endophthalmitis is still a major indication for enucleation or evisceration.

It is imperative to look for the means that may improve the chances of salvaging the eye after infectious endophthalmitis, therefore, the purpose of this study is to identify the risk factors associated with evisceration or enucleation in infectious endophthalmitis.

## Methods

This retrospective study was approved by the Institutional Review Board of Joint Shantou International Eye Center of Shantou University and the Chinese University of Hong Kong. We reviewed the medical records of patients who were diagnosed with endophthalmitis between January 2008 and September 2015 at the Joint Shantou International Eye Center of Shantou University and the Chinese University of Hong Kong. The data included age, sex, etiology, past medical history, clinical manifestations, microbiology, leukocyte counts and types of interventions were collected.

Chinese standard logarithm visual chart was used to measure initial VA and then converted into Logarithm of minimal angle of resolution (LogMAR) unit. The arbitrary LogMAR values for VA less than counting finger were used as follow: counting finger was converted to 2.0 LogMAR units, hand motion was converted to 2.3 LogMAR units, light perception was converted to 2.5 LogMAR units, and no light perception was converted to 3.0 LogMAR units^3^,^4^.

Two groups were divided according to whether the eyes underwent enucleation/evisceration or not. Independent sample t-test, Chi-square test, Fisher’s exact test, Mann-whitey test were used to analyze the difference between the two groups. Logistic regression analysis was used to detect the associated factors for enucleation or evisceration. The level of significance was defined as p-value less than 0.05. All the statistical analyses were performed with using SPSS software version 17.0 (SPSS Inc, Chicago, IL).

## Results

Our study included 210 eyes from 210 patients with endophthalmitis. The mean age was 44.6 ± 22.9 years (ranged from 5 to 93 years). There were 54 (25.7%) women and 156 (74.3%) men. 128 (61.0%) eyes had post-traumatic endophthalmitis, 42 (20.0%) had post-operative endophthalmitis, 23 (11.0%) had corneal ulcer related endophthalmitis, 17 (8.1%) had endogenous endophthalmitis. Among 210 eyes with endophthalmitis, thirty (14.3%) eyes were eviscerated or enucleated, other cases received vitrectomy with silicone oil (17.1%), vitrectomy without silicone oil (29.0%), intravitreal antibiotics (35.2%) and intravenous antibiotics (0.5%). Among 30 cases that received evisceration or enucleation, 4 eyes had received intravitreal antibiotics, 1 eye had received vitrectomy and 2 eyes had received conjunctival flap covering as initial treatment but these interventions failed to control the inflammation. None of the patients in the salvaging group received therapeutic keratoplasty. The rest of 23 eyes received evisceration or enucleation as initial treatment. The characteristics of patients who received evisceration or enucleation were listed in Table 1.

The mean age was older in the evisceration/enucleation group than in the salvaging group (58.7 ± 18.8 years vs. 42.3 ± 22.7 years, p < 0.001) (Table 2). There were more female in evisceration/enucleation group (43.3%) than in the salvaging group (22.2%) (p = 0.014). There was no statistical significant difference in laterality between the two groups.

Table 3 revealed that trauma-related endophthalmitis was significantly less frequent in the evisceration/enucleation group (26.7%) than in the salvaging group (67.8%) (p < 0.001). There were 6.7% cases with postoperative endophthalmitis in the evisceration/enucleation group compared with 22.2% in the salvaging group (p = 0.049). Corneal ulcer-related endophthalmitis was significantly more frequent in the evisceration/enucleation group (50.0%) than in the salvaging group (4.4%) (p < 0.001). Endogenous endophthalmitis was also significantly more frequent in the evisceration/enucleation group (23.3%) than in the salvaging group (5.6%) (p = 0.004).

Table 4 illustrated that the duration of onset was significantly longer in the evisceration/enucleation group (18.0 ± 29.4 days vs. 5.7 ± 12.2 days, p < 0.001) and the initial visual acuity was significantly worse (2.07 ± 0.64 vs. 2.7 ± 0.47, p < 0.001). There were no significant differences in the results of microbial culture, leukocyte counts, number of patients with diabetes, or duration of intravenous antibiotics between the two groups.

Logistic regression analysis revealed that after adjusting for confounding factors, corneal ulcer related endophthalmitis (b = 2.595 ± 0.639, p < 0.001), endogenous endophthalmitis (b = 1.878 ± 0.684, p = 0.006) and initial visual acuity (b = 3.135 ± 0.817, p < 0.001) were still significantly associated with evisceration or enucleation.

## Discussion

Our study found that corneal ulcer, endogenous endophthalmitis, female, older age, poor initial visual acuity and delayed intervention were strongly associated with evisceration or enucleation in univariate analysis. However, eyes with trauma-related endophthalmitis and postoperative endophthalmitis were less likely to be eviscerated or enucleated. Multivariate analysis showed that corneal ulcer, endogenous endophthalmitis and initial visual acuity were significantly associated with evisceration or enucleation after adjustment.

Tsai *et al*.^5^ reviewed 86 patients with endophthalmitis and found that evisceration or enucleation was performed in twenty patients (23.2%). In our study, only 14% of patients underwent evisceration or enucleation. This might be due to the advancement of medical technology for diagnosing and curing endophthalmitis. Similarly, Tsai *et al*. reported that poor initial visual acuity, older patients, corneal ulcer related endophthalmitis and endogenous endophthalmitis were significantly associated with evisceration and enucleation in univariate analysis^5^. In addition, we found that female and delayed intervention were risk factors for evisceration or enucleation. Trauma-related endophthalmitis and post-operation endophthalmitis were less likely to be evisceration or enucleation. In our multivariate analysis, corneal ulcer, endogenous endophthalmitis and initial visual acuity were significantly associated with evisceration or enucleation. This is the first study to report risk factors strongly associated with evisceration or enucleation using multivariate analysis with sufficient sample size.

In the literature, the high incidence of enucleation or evisceration among patients with corneal ulcer associated endophthalmitis has been reported. Henry *et al*. reported that 31% of patients with infectious keratitis-related endophthalmitis underwent enucleation or evisceration^6^. O’Neill *et al*. found that among 37 patients with microbial keratitis-associated endophthalmitis, 16 (43.3%) were eviscerated or enucleated as primary treatment, and 23 (62.2%) subsequently required evisceration or enucleation^7^. Scott IU *et al*. found that 3 (21.4%) out of 14 patients with corneal ulcer underwent evisceration or enucleation^8^. The risk factors for the progression from keratitis to endophthalmitis included: delayed diagnosis and treatment of microbial keratitis, the use of topical steroid, trauma, contact lens use and previous ocular surgical history^7^,^8^,^9^,^10^,^11^,^12^,^13^,^14^,^15^,^16^,^17^,^18^,^19^. In our study, 65.2% of corneal ulcer-related endophthalmitis required enucleation or evisceration which was higher than most previous reports. As the cornea may become opaque and thin in microbial keratitis, it may be difficult to perform vitrectomy unless combined with keratoplasty. Low socioeconomic status, poor accessibility to tertiary eye care, living in remote areas, lack of awareness of the adverse sequelae of keratitis and poor adherence to medical advice plausibly led to delayed intervention and failure to salvage the eyes among these patients. For instance, a few patients preferred evisceration for pain relief rather than other surgical attempts to save the eyeball due to the low cost and to avoid prolonged follow-up care. Further studies are required to identify the obstacles to prompt and optimal ophthalmic care in our locality to prevent the loss of any useful vision.

Endogenous endophthalmitis is generally associated with a number of systemic diseases, including liver abscess, pneumonia, endocarditis, urinary tract infection, meninges infection, diabetes mellitus, immunosuppression and some of them had a history of recent hospitalization or recent surgery^20^,^21^,^22^,^23^,^24^,^25^,^26^. Zenith *et al*. reported that 27.3% of eyes with endogenous endophthalmitis underwent enucleation or evisceration^27^. Sridhar *et al*. review 67 eyes with endogenous fungal endophthalmitis and found that patients with endogenous endophthalmitis caused by molds were more likely to be enucleated^22^. Eyes diagnosed with endogenous fungal endophthalmitis should be monitored closely, and they may require prompt vitrectomy for better outcome. Managing systemic comorbidities is crucial at the same time. Jackson *et al*. reviewed 342 cases of endogenous bacterial endophthalmitis, and found that 24% required evisceration or enucleation and both intravitreal dexamethasone and vitrectomy were associated with fewer evisceration or enucleations^26^. Our study also found that none of the endogenous endophthalmitis eyes that underwent vitrectomy required enucleation or evisceration. So vitrectomy was effective in saving the eye. In addition, 41% of eyes with endogenous endophthalmitis in our study were enucleated or eviscerated. Almost all of these patients had no useful vision when presented at our center, and were reluctant to follow medical advice on potential eye saving interventions including vitrectomy and keratoplasty.

In our study, both univariate analysis and multivariate analysis showed that poor initial visual acuity led to higher incidence of evisceration or enucleation for endophthalmitis, and 70% of patients who underwent evisceration or enucleation presented with no light perception. Poor initial visual acuity may be caused by the strong virulence of microorganism and/or delayed treatment of endophthalmitis, leading to the loss of the eye. Our results agreed with previous reports in the literature that poorer presenting visual acuity associated with worse prognosis. Gower *et al*. performed a population-based study in the United States and found that poor initial visual acuity was a risk factor for poor prognosis in patients with postoperative endophthalmitis^28^. Sallam *et al*. reviewed 44 eyes with candida endophthalmitis also reported that poor initial visual acuity significantly associated with worse visual outcome in both of their univariate analysis and multivariate analysis^29^.

According to Endophthalmitis Vitrectomy Study (EVS)^30^, the main treatment for endophthalmitis included intravitreal antibiotics and vitrectomy. Similar to previous reports, this study revealed that delayed diagnosis and delayed treatment of endophthalmitis have been associated with enucleation or evisceration^20^,^31^,^32^,^33^. Therefore, early diagnosis and intervention of endophthalmitis will improve the chance of salvaging the eye.

Our study also found that female, older age were risk factors for evisceration and enucleation. Many studies agree with the opinion that older age was risk factors for evisceration/enucleation. Gower *et al*. reported that older age was an important predictor of poor visual outcome^28^. Chiquet *et al*. and Ji *et al*. found that more virulent organisms were significantly associated with older age^28^,^34^. A meta-analysis also showed that old age was associated with higher risk of acute endophthalmitis^35^. Gaton *et al*. reported that enucleation or evisceration surgeries were more frequently performed in older age group^2^. In our series, female had higher risk for enucleation or evisceration. A possible explanation was that more women were diagnosed with keratitis associated endophthalmitis or endogenous endophthalmitis in our study. Gender was not an independent risk factor after adjusting for cofounders in multiple logistic regression analysis. With, However, some studies revealed that men were more likely to have endophthalmitis than female^1^,^35^. Further studies are required to investigate whether gender is associated with endophthalmitis and evisceration/enucleation.

This study is limited by its retrospective nature. The managements of patients were non-standardized, which may influence the final outcome. Furthermore, the sample in the evisceration/enucleation group was small compared with the salvaging group. Larger sample size will make the statistical analysis more valid. There are some cases that do not have microbial culture and some cases are culture negative. This reduces the sample size and we cannot determine which microbiological diagnoses are the risk factors for evisceration.

In summary, corneal ulcer, endogenous endophthalmitis, female, older age, poor initial visual acuity, delayed intervention were risk factors for evisceration or enucleation. Trauma-related endophthalmitis and postoperative endophthalmitis were less likely to be eviscerated or enucleated. After adjusting for confounding factors in multivariate analysis, corneal ulcer, endogenous endophthalmitis and poor initial visual acuity were strongly associated with evisceration or enucleation. Advocating for prompt referral to ophthalmologists, early intervention and closer monitoring of the disease progression in corneal ulcer are crucial for controlling ocular inflammation to prevent the loss of eyes.

## Additional Information

**How to cite this article**: Lu, X. *et al*. Risk factors for endophthalmitis requiring evisceration or enucleation. *Sci. Rep*. **6**, 28100; doi: 10.1038/srep28100 (2016).

## Figures and Tables

**Table 1:** Characteristics of patients with endophthalmitis who underwent evisceration or enucleation.

No.	Gender	Age (Years)	Cause	Microbial culture	Duration before intervention (days)	Initial treatment	Initial vision
1	Male	91	Corneal ulcer	Not done	3	Enucleation	NLP
2	Male	44	Post-trauma	Not done	1	Enucleation	NLP
3	Male	63	Endogenous	Not done	10	Enucleation	LP
4	Male	86	Corneal ulcer	Not done	30	Enucleation	NLP
5	Male	57	Corneal ulcer	Not done	30	Enucleation	LP
6	Female	90	Corneal ulcer	Not done	0.50	Enucleation	NLP
7	Male	49	Endogenous	Enterococcus faecium	2	IV Antibiotics	NLP
8	Male	58	Post-trauma	Not done	26	Enucleation	HM
9	Male	72	Corneal ulcer	Negative	5	Evisceration	LP
10	Female	39	Post-trauma	Not done	29	Enucleation	NLP
11	Male	31	Post-trauma	Negative	21	IV Antibiotics	NLP
12	Female	77	Corneal ulcer	Not done	120	Enucleation	NLP
13	Female	8	Corneal ulcer	Not done	3	Enucleation	NLP
14	Female	54	Corneal ulcer	Negative	0.50	Evisceration	NLP
15	Female	61	Endogenous	Negative	7	Evisceration	NLP
16	Female	58	Post-trauma	Staphylococcus capitis	4	Evisceration	NLP
17	Male	55	Corneal ulcer	Negative	28	IV Antibiotics	LP
18	Male	61	Corneal ulcer	Fungus	10	Conjunctival flap covering	LP
19	Female	61	Endogenous	Negative	120	Enucleation	NLP
20	Female	55	Endogenous	Staphylococcus epidermidis	1	Evisceration	NLP
21	Male	54	Post-trauma	Bacillus subtilis	1	Vitrectomy	NLP
22	Female	23	Corneal ulcer	Negative	14	Evisceration	NLP
23	Male	45	Endogenous	Negative	14	Evisceration	NLP
24	Male	67	Endogenous	Klebsiella pneumoniae	6	Evisceration	NLP
25	Female	79	Post-operation	Enterococcus faecalis	1	IV Antibiotics	HM
26	Female	56	Post-operation	Serratia marcescens	4	Evisceration	LP
27	Female	82	Corneal ulcer	Negative	7	Evisceration	NLP
28	Male	50	Corneal ulcer	Not done	15	Evisceration	NLP
29	Male	58	Corneal ulcer	Not done	13	Evisceration	NLP
30	Female	76	Corneal ulcer	Fungus	15	Conjunctival flap covering	NLP

IV: intravitreal; NLP: no light perception; LP: light perception; HM: hand movements.

**Table 2:** Comparison of characteristics between the patients with endophthalmitis receiving evisceration/enucleation or salvaging therapy.

	evisceration/enucleation	salvaging	p
N	30	180	
Age	58.7 ± 18.8	42.3 ± 22.7	<0.001#
Female	13 (43.3%)	40 (22.2%)	0.014+
Male	17 (56.7%)	140 (77.8%)	
Left eye	10 (33.3%)	83 (46.1%)	0.192+
Right eye	20 (66.7%)	97 (53.9%)	

^*^Fisher Text.

^#^Independent Sample t-test.

^+^Chi-square test.

**Table 3:** Comparison of the cause between the patients with endophthalmitis receiving evisceration/enucleation or salvaging therapy.

	evisceration/enucleation	salvaging	p
Trauma	6 (20.0%)	122 (67.8%)	<0.001+
Postoperative	2 (6.7%)	40 (22.2%)	0.049+
Corneal ulcer	15 (50.0%)	8 (4.4%)	<0.001*
Endogenous	7 (23.3%)	10 (5.6%)	0.004*

^*^Fisher text; ^#^Independent Sample t-test; ^+^Chi-square test.

**Table 4:** Comparison of clinical characters between the patients with endophthalmitis receiving evisceration/enucleation or salvaging therapy.

Factors	evisceration/enucleation	salvaging	p
N	30	180	
Diabetes	1 (3.4%)	6 (3.4%)	0.734*
Duration before intervention (days)	18.0 ± 29.4	5.7 ± 12.2	0.031#
Initial visual acuity			<0.001^−^
NLP	22 (73.3%)	13 (7.2%)	
LP	6 (20.0%)	31 (17.2%)	
HM	2 (6.7%)	78 (43.3%)	
FC or better	0 (0%)	58 (32.2%)	
Bacteria	7 (23.3%)	36 (20.0%)	0.599+
Fungus	2 (6.7%)	3 (1.1%)	0.099*
Leukocyte counts (*10^9^/L)	9.90 ± 3.64	10.98 ± 4.03	0.170#
Duration of intravenous antibiotics (days)	6.6 ± 4.9	6.2 ± 3.2	0.666#

^*^Fisher Text.

^#^Independent Sample t-test.

^+^Chi-square test; -: Mann-Whitney u text. NLP: no light perception; LP: light perception, HM: hand movement; FC: finger counting.
